# Nutraceuticals as Potential Radionuclide Decorporation Agents

**DOI:** 10.3390/nu13082545

**Published:** 2021-07-25

**Authors:** Vernieda B. Vergara, John F. Kalinich

**Affiliations:** Internal Contamination and Metal Toxicity Program, Armed Forces Radiobiology Research Institute, Uniformed Services University, Bethesda, MD 20889, USA; vernieda.vergara.ctr@usuhs.edu

**Keywords:** radionuclide, contamination, genistein, curcumin, quercetin, lentinan, chelation

## Abstract

Exposure of individuals to radioactive material as a result of ingestion of contaminated food and water is an increasing public health concern. Unfortunately, there are limited treatment modalities for dealing with these types of potentially toxic exposures. Recent research suggests that many plant-based nutraceuticals may possess metal-binding properties. This preliminary study investigated the ability of genistein, curcumin, quercetin, and lentinan to bind metals considered internal contamination risks, namely cesium, uranium, cobalt, and strontium, in a variety of matrices. The efficacy of these nutraceuticals in protecting cultured cells from metal-induced toxicity was also explored. Results showed that none of the compounds bound cesium or strontium. However, genistein, curcumin, and quercetin could bind uranium. Curcumin and quercetin also bound cobalt and could also protect cultured cells from metal-induced cytotoxicity. Lentinan did not bind any of the metals tested. Metal binding was also pH dependent, with no binding observed at lower pH values. This project showed that nutraceuticals could function as chelators for metals considered internal radionuclide contamination hazards. Further investigations are required in order to determine whether these compounds will become a new nontoxic arsenal of pharmaceutical compounds with which to treat radionuclide contamination.

## 1. Introduction

Ingestion of radionuclides by contaminated food and water is now becoming a more critical health threat as underscored by the Chernobyl [1,2,3] and Fukushima [4,5,6] nuclear disasters. Contamination events can occur from accidents at nuclear reactors, as well as from the use of a radiological dispersal device by terrorists, from the fallout as the result of the detonation of a nuclear weapon, or as a consequence of accidental or intentional poisoning of food sources or water supplies with radionuclides. The list of potential radiological contaminants can, at times, seem daunting. For example, a nuclear detonation can release over 400 radioactive isotopes. However, only about 40 of these are considered a potential hazard to human health primarily because of their long radiological half-lives or ability to concentrate in critical organ systems. Those rating a higher level of concern include cesium-137 (half-life 30.17 years), cobalt-60 (half-life 5.27 years), strontium-90 (half-life 28.9 years), and uranium-238 (half-life 4.47 × 10^9^ years). Ignoring prompt treatment from internal exposures can have just as devastating long-term effects on an individual as exposure from an external gamma source. Unfortunately, to date, only three compounds have been approved by the United States Food and Drug Administration for use in the decorporation of internalized radioisotopes and only for iodine, cesium, plutonium, and americium [7]. Potassium iodide was approved by the FDA in 1978 for use in preventing the uptake of radioactive iodine to the thyroid and is available for several pharmaceutical firms. In 2003, the FDA approved the use of Prussian Blue for internalized cesium-137. Prussian Blue is marketed by Heyl Pharmaceuticals under the trade name Radiogardase. In 2004, the FDA also approved the calcium and zinc salts of diethylenetriamine pentaacetic acid (DTPA) for use in enhancing the elimination of plutonium and americium from the body. Additionally, called pentetate, these compounds are manufactured by Hameln Pharmaceuticals. With limited choices available, there is a clear need for compounds of high specificity and low toxicity that can protect against the adverse effects of radiation exposure and accelerate the excretion of radionuclides from the body.

**Figure 1:**
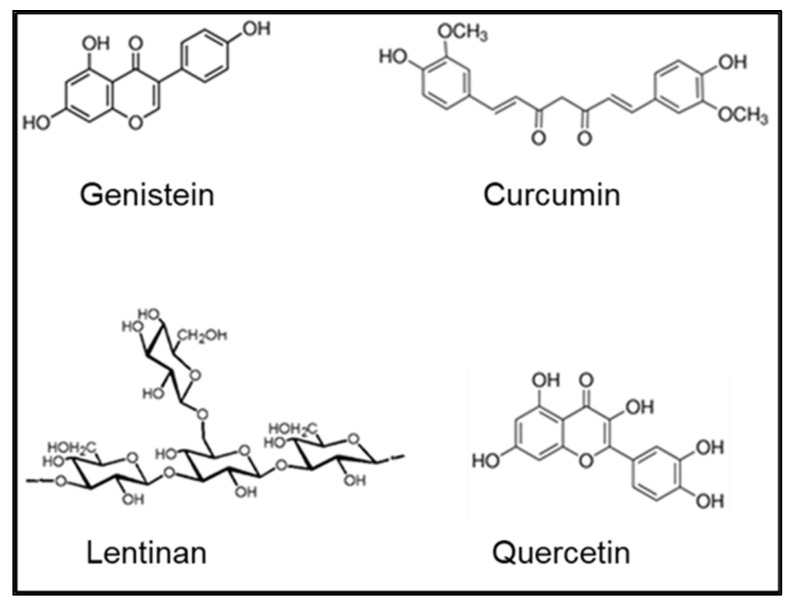
Chemical structures of tested nutraceuticals.

Although the health effects of internalized radionuclides are an area of major concern, treatments to safely address this issue are limited and can be loosely grouped into four general categories: uptake-reducing agents; blocking or diluting agents; mobilization agents; and chelating agents. While numerous categories of therapeutics have been proposed to prevent uptake or enhance the elimination of internal radionuclides, chelating agents remain the most promising avenue of treatment. Many of the proposed chelating agents for radionuclides were previously used as treatments for heavy-metal poisoning. Although successful in that regard, the use of chelating agents is not without concerns. Foremost being the inherent toxicity of the chelating agent itself. Recently there has been renewed interest in the use of natural products or nutraceuticals in the treatment of disease and the maintenance of health. Several of these products, including genistein, quercetin, and curcumin, as well as lentinan, a β-glucan compound derived from shiitake mushrooms, have also been proposed as nontoxic radiation countermeasures [8,9,10,11]. One of the lesser investigated properties of these compounds is the ability to bind metals, most likely through the structural hydroxyl moieties (Figure 1). Reports in the literature have shown that genistein is capable of binding copper and iron [12,13], quercetin can chelate copper and lead [14,15], curcumin can bind cadmium and lead [16], and lentinan can chelate cadmium [17]. Despite these findings, there have been no reports on the ability of these compounds to bind those metals that are of concern with respect to internal contamination, namely, cesium, strontium, uranium, and cobalt. This report represents the initial effort to address those shortcomings. If future investigations using an animal model prove successful, the result could be a nontoxic arsenal of pharmaceutical compounds with which to treat internal radionuclide contamination. An additional benefit is that most of these plant-based nutraceuticals have also been shown to be useful in protecting against the deleterious effects of external radiation exposure [8,9,10,11], an effect attributed primarily to the antioxidant properties of the compounds. Thus, if these compounds prove to be effective chelators, there is the potential added bonus of antioxidant protection against free radical damage induced by the internalized radionuclides. However, the lack of a knowledge base with respect to the metal chelating ability of these compounds, particularly with metals considered likely internal contamination candidates, necessitated this initial investigation to be conducted using in vitro techniques to determine if further studies in an animal model are warranted.

## 2. Materials and Methods

### 2.1. Materials

All products used in this study were commercially available in high-purity formulations. Genistein, quercetin, and curcumin were purchased from Sigma-Aldrich Chemical (St. Louis, MO, USA), while lentinan was obtained from GlycaNova (Fredrikstad, NOR). Test metals were purchased from Sigma-Aldrich or from Alfa Aesar (Ward Hill, MA, USA) and were of the highest purity available. With the exception of uranium, all other test metals were nonradioactive to reduce personnel exposure and eliminate the production of radioactive waste. All other laboratory chemicals were purchased from Sigma-Aldrich or Fisher Scientific (Pittsburgh, PA, USA) and were of the highest quality available.

### 2.2. Assessment of Metal Binding

UV/visible spectra were obtained using an Agilent Model G1103A Spectrophotometer (Santa Clara, CA, USA). Due to the multiple ring structures of the nutraceuticals, numerous peaks are observed in the UV/visible spectra. Binding of metal results in a modification of the spectrum, usually a change in the λ_max_ and a hypsochromic or bathochromic shift of the spectrum [9,10]. Concentrations ranging from 0 to 100 µM were tested for both the nutraceutical compounds and the metals. The binding characteristics of genistein, quercetin, curcumin, and lentinan were assessed for cesium, cobalt, uranium, and strontium in a variety of biofluids and water. Simulated biofluids, including serum, lung, gastric, and intestinal were utilized and prepared as previously described [18].

### 2.3. Cell Culture

J774, a murine macrophage cell line, and Caco-2, a human intestinal epithelial cell line, were utilized to determine if chelation with nutraceuticals can reduce or eliminate the cytotoxicity associated with those metals considered likely internal contamination threats. Both cell lines were purchased from the American Type Culture Collection (Manassas, VA, USA). All cell culture reagents were purchased from Invitrogen (Grand Island, NY, USA). J774 cells were grown in RPMI-1640 medium supplemented with 10% fetal bovine serum [19] while Caco-2 cells were cultured in F12-K medium supplemented with 20% fetal bovine serum [20]. Stock cultures were maintained in tissue culture flasks (75 cm^2^ area) in a humidified atmosphere of 5% CO_2_/95% air. For both cell lines, the medium was replaced every 2 to 3 days and cells were subcultured when 70–80% confluent based on direct microscopic observation. Cell numbers and viability assessments were conducted using trypan blue (Gibco) dye exclusion with the Bio-Rad Model TC-20 Cell Counter (Hercules, CA, USA).

### 2.4. MTT Assay

Cells were plated onto 96-well plates at 10,000 cells/well (Caco-2) or 5000 cells/well (J774) at 100 μL per well, and allowed to incubate at 37 °C, 5% CO_2_ for 24 h. Media were then removed, and cells were treated with the appropriate media spiked with metal or nutraceutical compound in a serial dose dilution ranging from 0 μM to 1000 μM and incubated for an additional 24 h prior to viability assays. The CellTiter 96^®^ Aqueous One Solution Cell Proliferation Assay kit (Promega Corporation, Madison, WI, USA) was used to assess metabolic viability. The colorimetric assay is based upon the ability of dehydrogenase enzyme systems, located in the cell mitochondria, to reduce a tetrazolium compound to a colored formazan product. Briefly, after the 24 h treatment incubation period, 10 µL of CellTiter 96^®^ Aqueous One Solution Reagent was added to each plate well, incubated 1 h, then absorbance determined at 490 nm using a microplate reader (SpectraMax Model 250 Microplate Spectrophotometer, Molecular Devices Corporation, Sunnyvale, CA, USA). The metabolic viability of the treated cells was compared to the media-only control cells.

### 2.5. Microscopy

For microscopy, cells were plated at 50,000 cells/chamber on Nunc Lab-Tek II 8-chamber slides (Fisher Scientific, Pittsburgh, PA, USA) and treated in the same manner as the 96-well plates. The medium was aspirated, the chambers washed with Dulbecco’s PBS (DPBS, Gibco), and cells were fixed for 5 min with ice-cold methanol. After removing the methanol, the slides were air-dried and then stained with Giemsa (Gibco) stain (1:20 dilution of stock) for 10 min followed by extensive washing with tap water. Images were taken with an Olympus BX61 Microscope equipped with a D72 camera and processed with cellSens Entry Software (version 1.5) (Olympus America, Melville, NY, USA).

### 2.6. ICP-MS Metal Measurements

The metal content of the collected samples was determined using an inductively coupled plasma–mass spectrometer (XSeries 2 ICP-MS, ThermoElectron North America, LLC., Madison, WI, USA) equipped with a Cetac ASX520 Autosampler (Cetac Technologies, Omaha, NE, USA). The plasma gas was high-purity (99.997%) liquid argon. Instrument calibration was accomplished using the appropriate metal standards (SPEX CertiPrep, Metuchen, NJ, USA) in 2% nitric acid (Ultrapure Optima Grade, Fisher Scientific, Newark, DE, USA). Metal concentration levels were obtained by reference to the slope of the calibration curve (counts per second/ng per milliliter) and an internal standard.

Samples were processed using a digestion procedure with nitric acid (Ultrapure Optima Grade) and hydrogen peroxide (Semi-Conductor Grade, Sigma-Aldrich) as previously described [21]. Prior to analysis, the samples were resuspended in 2% nitric acid and analyzed as described above. Limit of Detection/Limit of Quantitation, in ppb, are as follows: Co—0.03/0.06; Cs—0.01/0.02; Sr—0.01/0.05; U—0.02/0.07.

### 2.7. Statistical Analysis

Statistical analyses were performed using GraphPad Prism Software (version 9.0.2, La Jolla, CA, USA). Unpaired t-tests were performed, with specific group comparisons or any other statistical analyses noted. *p* values less than 0.05 were considered statistically significant.

## 3. Results

Solubility assessments indicated that, except for lentinan, all nutraceutical test compounds were soluble in DMSO. Lentinan was soluble in water. None of the compounds were soluble in ethanol. Therefore, concentrated stock solutions were prepared using DMSO, while lentinan was prepared in water. After dilution into the appropriate medium, final DMSO concentrations were 0.1% or less. Stock solutions of the nutraceuticals were found to be stable at room temperature for over 2 months, based upon UV/visible spectroscopy. Compounds were also stable within pH ranges of 4.3 to 7.4, as determined spectrophotometrically, using the following buffers at 100 mM final concentration: acetate (pH 4.3), MES (pH 5.5), and phosphate (pH 7.4).

**Figure 2:**
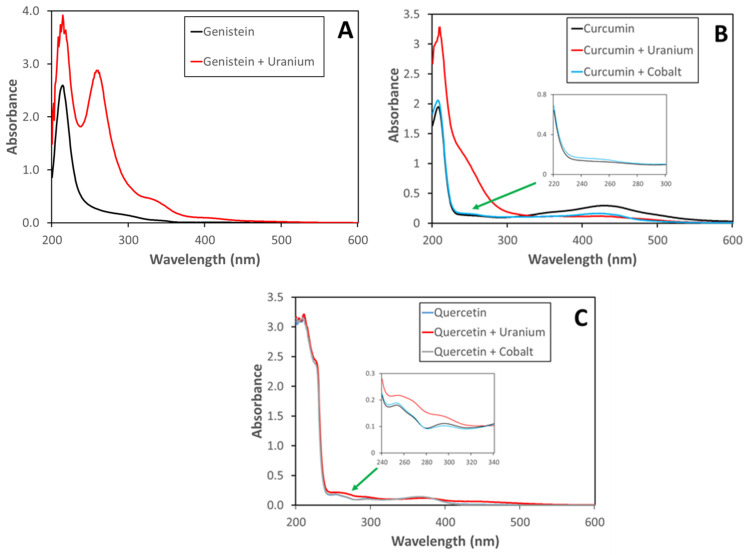
UV–visible spectra of metals and nutraceuticals in phosphate buffer (pH 7.4): (**A**) genistein (10 µM) with uranium (10 µM); (**B**) curcumin (10 µM) with uranium (10 µM) or cobalt (10 µM); (**C**) quercetin (10 µM) with uranium (10 µM) or cobalt (10 µM).

Compounds were initially tested for their ability to bind metals, first in a water matrix, then in buffer solutions, and finally simulated biofluids. The following metals were tested: cobalt, strontium, cesium, and uranium. No compound was found to bind all of the test metals. Lentinan did not bind any metals and was dropped from further evaluation. As seen in Figure 2A, genistein bound to uranium, resulting in a large peak at approximately 260 nm and a shoulder at 335 nm. Genistein did not bind cesium, strontium, or cobalt. Curcumin bound uranium (251 nm) and, to a lesser extent, cobalt (249 nm) (Figure 2B) but not cesium or strontium. Quercetin (Figure 2C) also bound uranium (255 nm) and cobalt (253 nm) but not cesium or strontium. All compounds gave similar binding results when tested in water, acetate buffer (pH 4.3), MES buffer (pH 5.5), or phosphate buffer (pH 7.4). Similar metal-binding patterns were also observed using simulated serum, lung, or intestinal biofluids. However, no metal binding was observed in simulated gastric fluid, most likely due to the low pH (pH < 2) of the simulated fluid. Of the metals of concern with respect to ingested radionuclide exposure, cesium, and strontium were not strongly chelated by any of the nutraceuticals tested. Using the Beer–Lambert Law, the molar absorption coefficient can be determined for the tested nutraceuticals and metal complexes. These results are shown in Table 1.

**Figure 3:**
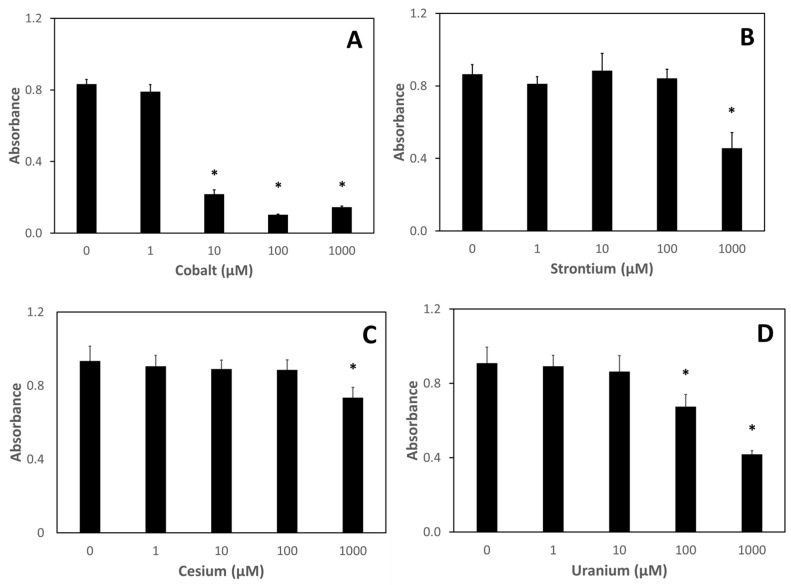
Metabolic viability of J774 cells after treatment with metals. J774 cells were treated with various concentrations of metals for 24 h after which metabolic viability was assessed using the MTT assay: (**A**) cobalt; (**B**) strontium; (**C**) cesium; (**D**) uranium. Data are the mean of three independent experiments. Error bars represent standard deviation. An * denotes a result statistically different from the no metal control as determined by unpaired t-test with significance at *p* < 0.05.

To determine whether nutraceuticals could ameliorate metal-induced damage, toxicity assessments of cesium, strontium, cobalt, and uranium on two mammalian cell lines were conducted. The effect of a 24 h treatment of various concentrations of the test metals on the metabolic viability of J774 cells, a murine macrophage cell line, is shown in Figure 3. Except for the highest concentration tested (1000 µM), neither cesium nor strontium significantly affected metabolic viability (Figure 3B,C). A dose–response was observed with uranium-treated J774 cells starting at 100 µM uranium (Figure 3D). Surprisingly, cobalt exposure of J774 cells resulting in a significant decrease of metabolic viability at concentrations as low as 10 µM (Figure 3A).

**Figure 4:**
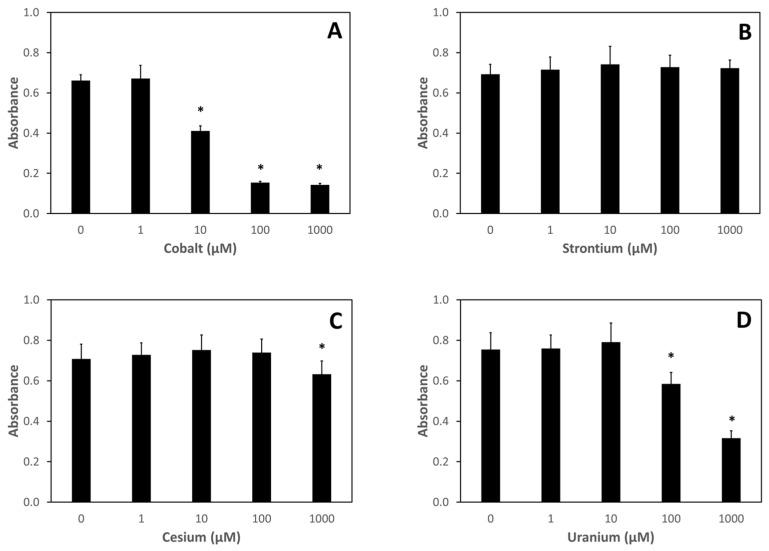
**Metabolic Viability of Caco-2 Cells after Treatment with Metals.** Caco-2 cells were treated with various concentrations of metals for 24 h after which metabolic viability was assessed using the MTT assay: (**A**) cobalt; (**B**) strontium; (**C**) cesium; (**D**) uranium. Data are the mean of three independent experiments. Error bars represent standard deviation. An * denotes a result statistically different from the no metal control as determined by unpaired t-test with significance at *p* < 0.05.

The metal toxicity results from Caco-2 cells, a human intestinal epithelial line, are shown in Figure 4. Similar findings are seen for metal effects on metabolic viability for the Caco-2 cells, as were seen for the J774 cells. Cesium and strontium had little if any effect on metabolic viability (Figure 4B,C). Uranium treatment affected Caco-2 metabolic viability at concentrations 100 µM and higher (Figure 4D), while cobalt significantly affected metabolic viability at concentrations as low as 10 µM (Figure 4A).

**Figure 5:**
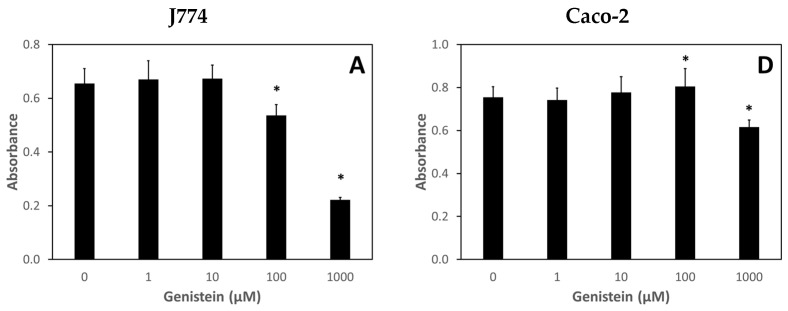

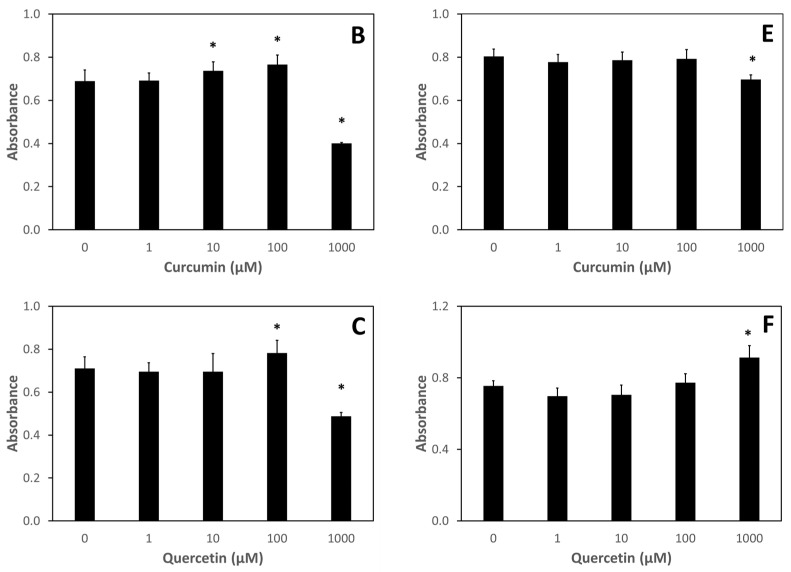
Effect of nutraceutical exposure on metabolic viability of J774 and Caco-2 cells. Cells were treated with various concentrations of nutraceuticals for 24 h after which metabolic viability was assessed using the MTT assay: (**A**,**D**) J774 and Caco-2 cells, respectively, with genistein; (**B**,**E**) J774 and Caco-2 cells, respectively, with curcumin; (**C**,**F**) J774 and Caco-2 cells, respectively, with quercetin. Data are the mean of three independent experiments. Error bars represent standard deviation. An * denotes a result statistically different from the no metal control as determined by unpaired t-test with significance at *p* < 0.05.

Assessment of nutraceutical exposure on the viability of J774 and Caco-2 cells is shown in Figure 5. Except for the highest concentration tested, 1000 μM, nutraceutical treatments were nontoxic from a metabolic viability standpoint. Genistein treatment of J774 cells at a concentration of 100 μM for 24 h did result in a slight but statistically significant decrease in metabolic viability.

Nutraceutical treatment also did not greatly affect the morphology of either J774 or Caco-2 cells (Supplemental Figures S1 and S2).

**Figure 6:**
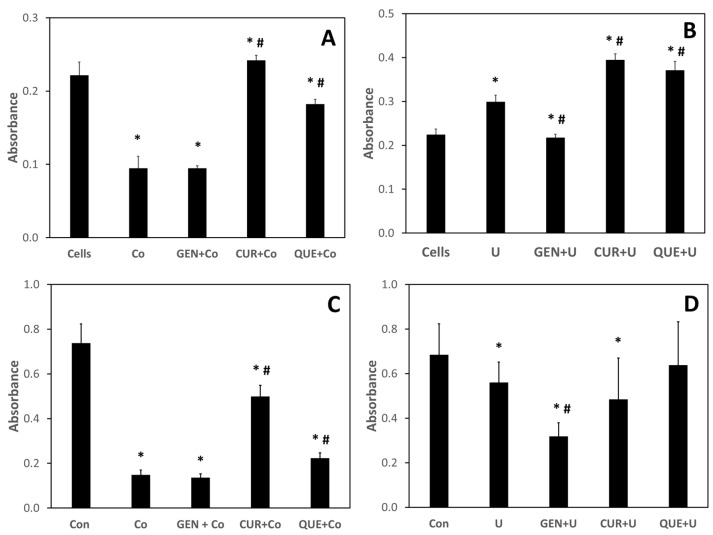
The ability of nutraceutical treatment to rescue cells from metal-induced metabolic toxicity. Effect of nutraceutical exposure on metabolic viability of J774 and Caco-2 cells. Cells were treated with cobalt (Co) or uranium (U) at 100 μM for 1 h followed by the addition, at 100 μM final concentration, of genistein (GEN), curcumin (CUR), or quercetin (QUE). After a 24 h incubation, metabolic viability was assessed using the MTT assay: (**A**,**B**) J774 cells treated with cobalt or uranium, respectively; (**C**,**D**) Caco-2 cells treated with cobalt or uranium, respectively. Data are the mean of three independent experiments. Error bars represent standard deviation. An * denotes a result statistically different from control and a # represents a statistically significant difference from the metal-treated cells, as determined by unpaired t-test with significance at *p* < 0.05.

To determine if nutraceutical treatment could ameliorate metal-induced metabolic toxicity, J774 and Caco-2 cells were treated with 100 μM of either cobalt or uranium, followed 1 h later by the addition of genistein, curcumin, or quercetin to a final concentration of 100 μM. Metabolic viability was assessed after 24 h using the MTT assay. As none of the nutraceuticals tested bound cesium or strontium, and cesium and strontium were relatively nontoxic in both J774 and Caco-2, they were not tested in this experiment. As seen in Figure 6, both treatments with either curcumin or quercetin prevented the cobalt-induced decrease of metabolic viability in both J774 and Caco-2 cells. Somewhat surprisingly, genistein treatment had no effect on either cobalt- or uranium-induced metabolic viability decreases in either J774 or Caco-2 cells.

Finally, because of the demonstrated phagocytotic capability of J774 cells, the ability of the cells to internalize cobalt and uranium was assessed, as was the ability of the nutraceuticals to affect this import. Table 2 shows cellular metal levels after a 30 min incubation with metal (100 μM) with and without nutraceutical (100 μM). Cells were then harvested, counted, and prepared for metal analysis via inductively coupled plasma–mass spectrometry. While elevated levels of cobalt are found in J774 cells, only quercetin treatment significantly increased the intracellular levels compared to control. On the other hand, both quercetin and curcumin significantly enhanced uranium uptake by J774 cells.

## 4. Discussion

Internal radionuclide exposure as a result of ingestion of contaminated food and water is becoming an increasing public health concern. However, medical treatment options are limited [22]. Recently there has been renewed interest in the use of nutraceuticals in the treatment of disease and the maintenance of health. Several of these have also been proposed for use as nontoxic radiation protection countermeasures. One of the lesser investigated properties of these compounds is the ability to bind metals, most likely through structural hydroxyl moieties. Reports in the literature have shown that genistein is capable of binding copper and iron [12,13], quercetin can chelate copper and lead [14,15], curcumin can bind cadmium, and lead [16], and lentinan can chelate cadmium [17]. Despite these findings, there have been no reports on the ability of these compounds to bind those metals that are of concern with respect to internal contamination. Of the many potential internal contaminants, cesium, strontium, cobalt, and uranium are considered the most likely [7]. This communication assessed the ability of several nutraceuticals to bind to likely internal contaminants as well as rescue cultured mammalian cells from metal-induced decreases in metabolic viability.

Using UV–visible spectroscopy, we found that genistein bound uranium with a resulting bathochromic shift of the spectrum. Genistein did not bind cobalt, strontium, or cesium. Curcumin and genistein also bound uranium and cobalt. Changes in the UV–visible spectra were very minor, compared to those seen with genistein and uranium. As with genistein, neither curcumin nor quercetin was found to bind to cesium or strontium. Lentinan did not bind any of the metals tested and was eliminated from further testing. For those nutraceuticals that bound metals, binding was possible in a wide range of matrices and pH conditions. We found that binding was possible in pH ranges from 4.3 to 7.4 in buffer and simulated biofluids such as serum, lung, and intestine. No metal binding was observed at low pH (<2) such as the conditions found in gastric fluid.

To assess whether nutraceutical treatment could rescue mammalian cells from metal-induced decreases in metabolic viability, we first investigated the toxicity of metal exposure alone on both J774 cells, a murine macrophage line, and Caco-2 cells, a human intestinal epithelial line. Using the MTT metabolic viability assay, we found that cesium and strontium treatment exhibited cytotoxicity only at the highest concentration tested (1000 μM). On the other hand, both cobalt and uranium exposures resulted in decreased metabolic viability of both cell lines at much lower concentrations, with significant decreases seen at 100 μM for uranium and 10 μM for cobalt in both cell lines. Whether these effects on metabolic viability are the result of metal-induced free radical reactions remains to be investigated. Both uranium and cobalt have been shown to aid in the production of reactive oxygen and nitrogen species [23,24,25,26,27,28]. To our knowledge, no such properties have been ascribed to either cesium or strontium.

Toxicity assessments of the nutraceutical compounds were also determined using the MTT metabolic viability assay. Again, only the highest concentrations (1000 μM) tested demonstrated any significant cytotoxicity. Genistein at 100 μM also resulted in a decrease in metabolic viability but only for J774 cells. Conversely, in some cases, nutraceutical treatment enhanced metabolic viability in both J774 and Caco-2 cells. Although lentinan was not found to bind any of the metals tested, it was found to be nontoxic to J774 and Caco-2 cells at all concentrations tested (data not shown).

Using both J774 and Caco-2 cells, we investigated whether the nutraceuticals could rescue cobalt- and uranium-exposed cells from decreases in metabolic viability. Both curcumin and quercetin were able to ameliorate the adverse effects of cobalt treatment in both cell lines, with curcumin treatment being more effective than quercetin. Somewhat surprisingly, genistein treatment had no effect on the cobalt-induced decrease in metabolic viability in either cell line. Curcumin and quercetin were also able to rescue J774 cells from uranium-induced metabolic viability decreases in J774 cells, with genistein again having no effect. None of the nutraceuticals were effective in preventing uranium-induced metabolic viability decreases in Caco-2 cells. It is not known whether the “rescue effect” is due to the metal-binding capabilities of the tested nutraceutical or their reported antioxidant properties [29,30,31]. In addition, the ability of the nutraceuticals to induce the expression of proteins that may function in the protection and/or repair of metal-induced damage cannot be overlooked. For example, genistein has been shown to induce the expression of metallothionein, a metal-binding protein with antioxidant properties, in Caco-2 cells [32]. Further investigations are required to decipher this mechanistic question.

To determine whether nutraceutical treatment affected metal translocation from the extracellular medium to the interior of the cells, J774 and Caco-2 cells were treated with cobalt or uranium, followed by genistein, curcumin, or quercetin. The cells were then processed, and metal content was analyzed by inductively coupled plasma–mass spectrometry. Cobalt and uranium levels in Caco-2 cells were below the limit of detection. However, both cobalt and uranium were taken up by J774 cells, a cell type with a known phagocytotic ability with respect to some metals [33]. Genistein and curcumin did not enhance cobalt uptake by the J774 cells; however, quercetin treatment increased cobalt uptake by 3–4-fold. Opposite results were found with uranium. Although uranium was only taken up by J774 cells at extremely low levels, most likely due to the short exposure times, treatment with either genistein or curcumin significantly increased uptake. Quercetin treatment did not affect uranium uptake in J774 cells.

While research continues by others on developing treatments for internal radionuclide exposure [34,35,36,37], the results of this preliminary investigation showed that nutraceutical compounds could function as chelators for some metals considered internal radionuclide contamination hazards. Clearly, further research is required. Since these toxic exposures are due to ingestion, an important advance will be to modify the nutraceutical compound to keep it localized in the gastrointestinal tract. Due to the hydroxyl moieties on the tested nutraceuticals, it should be possible to chemically link them to a nonsoluble substrate (e.g., silica) of sufficient size to ensure the complex remains localized to the gastrointestinal tract and excreted with the bound metal in the feces. There are several rodent models that are currently in use that would be useful in testing the efficacy of the nutraceuticals in eliminating internalized radionuclides [38,39,40,41,42]. However, since many of these nutraceuticals can also bind metals essential for good health [12,13,14,43], this suggests that if these nutraceuticals are used in elevated doses to enhance the elimination of internal radionuclide contaminants from the body, administrating mineral supplements may be required to maintain normal metal homeostasis. Finally, with further investigations, it might be possible to provide a nontoxic arsenal of pharmaceutical compounds with which to treat internal radionuclide contamination. An additional benefit is that all of these nutraceuticals have also been shown to be useful in protecting against the deleterious effects of external radiation exposure, an effect attributed primarily to the antioxidant properties of the compounds. Thus, if these compounds prove to be effective metal chelators in vivo, there is the potential added bonus of antioxidant protection against free radical damage induced by the internalized radionuclides.

## Figures and Tables

**Table 1:** Molar absorption coefficients of nutraceuticals and metal complexes.

	ε (M^−1^ cm^−1^)
Genistein (260 nm)	25,400
Genistein + Uranium	288,000
Curcumin (249 nm)	13,300
Curcumin + Cobalt	15,800
Curcumin (251 nm)	13,100
Curcumin + Uranium	95,300
Quercetin (253 nm)	18,100
Quercetin + Cobalt	18,900
Quercetin (255 nm)	17,900
Quercetin + Uranium	21,800

**Table 2:** Metal uptake by J774 cells in the absence and presence of nutraceuticals.

	ng Metal/10^5^ Cells
Cobalt	994.12 ± 40.28
Cobalt + Genistein	1215.31 ± 119.82
Cobalt + Curcumin	824.00 ± 68.82
Cobalt + Quercetin	3726.60 ± 143.48 *
Uranium	0.69 ± 0.13
Uranium + Genistein	11.45 ± 3.19 *
Uranium + Curcumin	14.59 ± 1.50 *
Uranium + Quercetin	0.84 ± 0.11

Data are the mean of three independent experiments with three replicates per experiment and metal content normalized to 10^5^ cells. Errors are standard deviations. An * denotes a result statistically different from the metal-alone control as determined by unpaired t-test with significance at *p* < 0.05.

## Data Availability

All data supporting the results described are provided within the manuscript. Any unreported data can be obtained by contacting the corresponding author.

## Supplementary Materials

The following are available online at https://www.mdpi.com/article/10.3390/nu13082545/s1, Figure S1: Nutraceutical-treated J774 cells, Figure S2: Nutraceutical-treated Caco-2 cells, Table S1: Inductively coupled plasma–mass spectrometer parameters.
