# Supplementary material for: Nutraceuticals as Potential Radionuclide Decorporation Agents
Source: Nutrients. 2021 Jul 25;13(8):2545. doi: 10.3390/nu13082545 (PMC8400047; doi:10.3390/nu13082545)
Supplement: Supplementary file 1 [file nutrients-13-02545-s001.zip › nutrients-1291212-supplementary.pdf]

Supplemental Figure S1: Nutraceutical-Treated J774 Cells

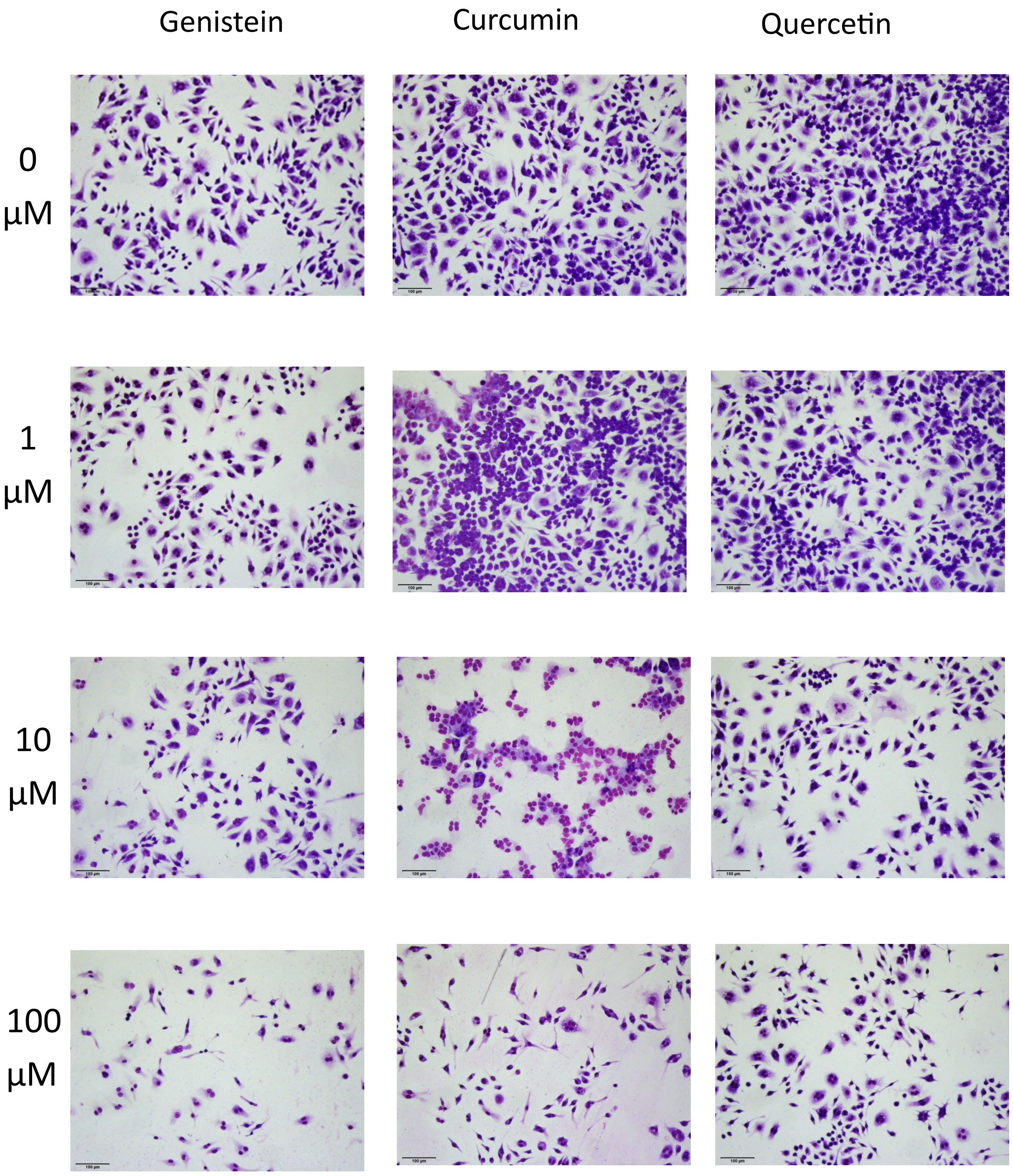

Cells were treated with the indicated nutraceutical at the indicated concentration for 24h, fixed with methanol, and stained with Giemsa. Scale bar is 100 µm.

Supplemental Figure S2: Nutraceutical-Treated Caco-2 Cells

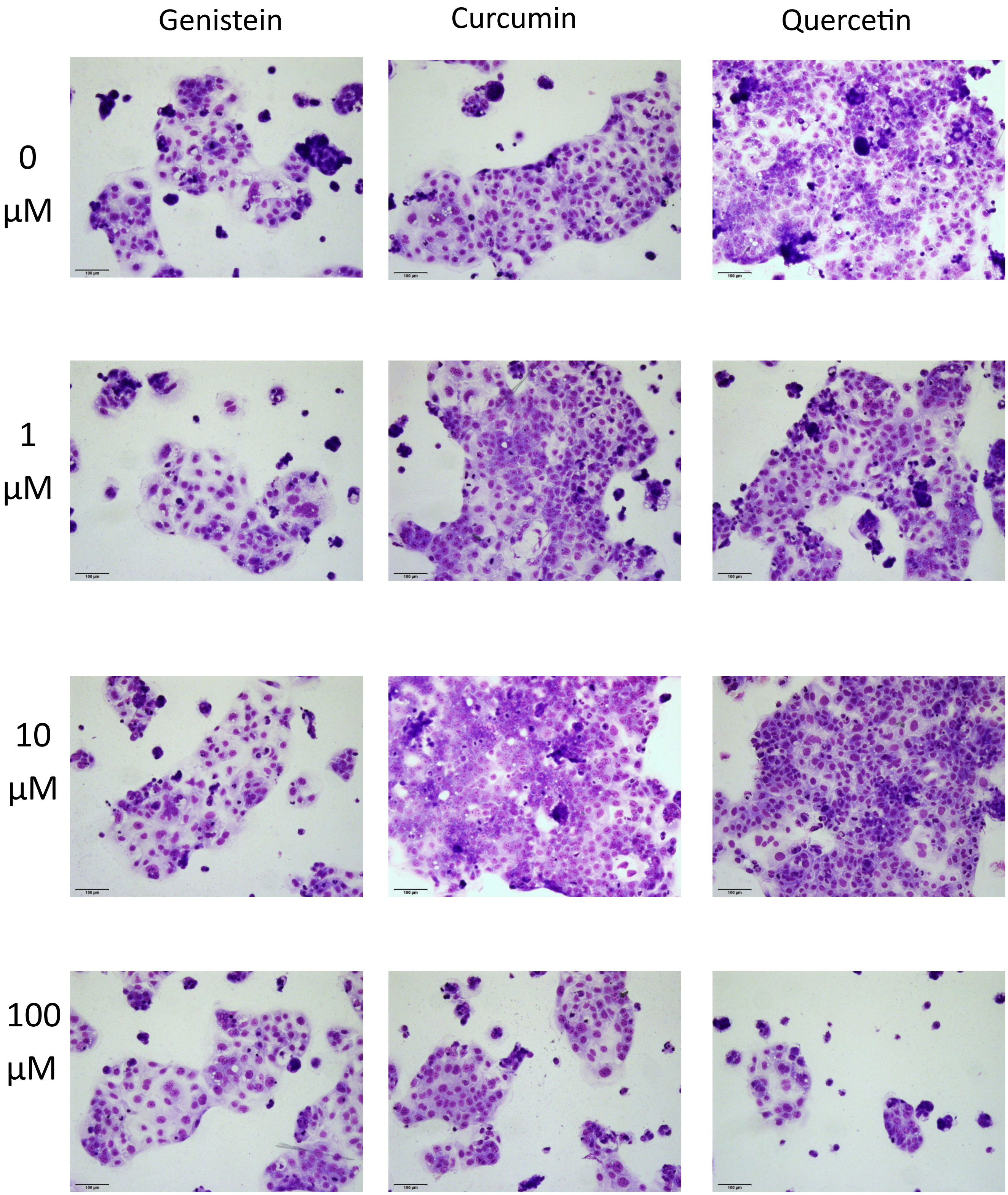

Cells were treated with the indicated nutraceutical at the indicated concentration for 24h, fixed with methanol, and stained with Giemsa. Scale bar is 100 µm.

Table S1
Inductively Coupled Plasma-Mass Spectrometry Operating Conditions and Parameters

Instrument Parameters	
Nebulizer type	Concentric
Spray chamber	Conical, with impact bead
Sampler cone	Platinum, 1mm orifice diameter
Skimmer cone	Platinum, 0.7 mm orifice diameter
Sample uptake rate	1.0 ml/min
Sample read delay	45 sec
Plasma conditions	
RF power	1400 W
Plasma argon gas flow	13.0 L/min
Auxiliary argon gas flow	0.80 L/min
Nebulizer gas flow	0.91 L/min
Mass spectrometer settings	
Scanning mode	Peak jump
Sweeps	100
Dwell time	500 μs
Channels/mass	1
Acquisition time	10 sec
Number of readings/replicate	3
Number of replicates	2
